# Incidence of Kaposi Sarcoma in HIV-infected patients – a prospective multi-cohort study from Southern Africa

**DOI:** 10.1186/1750-9378-7-S1-O20

**Published:** 2012-04-19

**Authors:** Julia Bohlius, Fabio Valeri, Mhairi Maskew, Hans Prozesky, Cleophas Chimbetete, Priscilla Lumano-Mulenga, Daniela Garone, Thomas Gsponer, Matthias Egger

**Affiliations:** 1 Institute for Social and Preventive Medicine, University of Bern, Bern, Switzerland; 2 Health Economics and Epidemiology Research Office, Department of Medicine, University of Witwatersrand, Johannesburg, South Africa; 3 Division of Infectious Diseases, Department of Medicine, University of Stellenbosch, Cape Town, South Africa; 4 Newlands Clinic, Harare, Zimbabwe; 5 Centre for Infectious Diseases and Research in Zambia, Lusaka, Zambia; 6 Medecins Sans Frontieres, South Africa

## Background

The incidence of Kaposi Sarcoma (KS) is high in sub-Saharan Africa. Data on KS among HIV-infected patients receiving and not yet receiving antiretroviral therapy (ART) are, however, scarce in Africa. Within the framework of a large multi-cohort project, the International epidemiologic Database to Evaluate AIDS (IeDEA), we estimate the incidence and risk factors for the development of KS in HIV-infected patients in Southern Africa.

## Methods

We analyzed prospectively collected data of HIV-infected children and adults participating in IeDEA-SA. We included all patients who were ART naive at start of observation, regardless of cancer history, with at least 30 days follow up. Prevalent KS cases were also excluded. Incidence rates and 95% confidence intervals (CI) were calculated based on the Poisson distribution; risk factors were estimated using crude and adjusted Cox proportional hazard models. Hazard ratios (HR) with 95% CI and medians with interquartile ranges (IQR) are presented.

## Results

We included 184,592 patients from 10 cohort studies in Botswana, Mozambique, South Africa, Zambia and Zimbabwe. The median age was 34 years (IQR 28–41), the median CD4 cell count at first contact was 152 cells/µl (IQR 75-252) and 146 cells/µl (IQR 74-226) at start of ART. 61% of patients were female. During a total follow-time of 391,852 person-years, 349 patients developed KS before starting ART, 585 developed KS after starting ART and 183,658 remained KS-free. In patients not receiving ART the KS incidence rate was 624 (95% CI 562–692) per 100,000 person-years and in patients receiving ART the KS incidence rate was 174 (95% CI 161-189) per 100,000 person-years, rate ratio for ART versus no ART = 0.28 (95% CI 0.24 - 0.32). Univariate and multivariate analyses showed that men were more likely than women to develop KS and that the incidence rate for KS increased with increasing age and with decreasing CD4 cell counts. These effects were more pronounced in patients not receiving ART than in patients receiving ART.

## Conclusions

In Southern African countries with a high prevalence of HHV-8 the risk of developing KS in HIV infected patients receiving ART increases steeply with age and immune-suppression. ART reduced the incidence of KS substantially.

